# β-hydroxybutyrate resensitizes colorectal cancer cells to oxaliplatin by suppressing H3K79 methylation in vitro and in vivo

**DOI:** 10.1186/s10020-024-00864-1

**Published:** 2024-06-23

**Authors:** Meng Deng, Peijie Yan, Hui Gong, Guiqiu Li, Jianjie Wang

**Affiliations:** 1 School of Basic Medicine, Jiamusi University, No. 258 Xuefu Street, Jiamusi, 154007 Heilongjiang Province China; 2 The Heilongjiang Hospital of Beijing Children’s Hospital, Capital Medical University, Harbin, 150000 China; 3 Clinical Laboratory, Huazhong University of Science and Technology Union Shenzhen Hospital and the Affiliated Shenzhen Sixth Hospital of Shenzhen University, No. 89 Taoyuan road, Nanshan district, Shenzhen, 518052 China

**Keywords:** Colorectal cancer, Β-hydroxybutyrate, Oxaliplatin, Drug resistance, H3K79me

## Abstract

**Background:**

Ketone β-hydroxybutyrate (BHB) has been reported to prevent tumor cell proliferation and improve drug resistance. However, the effectiveness of BHB in oxaliplatin (Oxa)-resistant colorectal cancer (CRC) and the underlying mechanism still require further proof.

**Methods:**

CRC-Oxa-resistant strains were established by increasing concentrations of CRC cells to Oxa. CRC-Oxa cell proliferation, apoptosis, invasion, migration, and epithelial-mesenchymal transition (EMT) were checked following BHB intervention in vitro. The subcutaneous and metastasis models were established to assess the effects of BHB on the growth and metastasis of CRC-Oxa in vivo. Eight Oxa responders and seven nonresponders with CRC were enrolled in the study. Then, the serum BHB level and H3K79me, H3K27ac, H3K14ac, and H3K9me levels in tissues were detected. DOT1L (H3K79me methyltransferase) gene knockdown or GNE-049 (H3K27ac inhibitor) use was applied to analyze further whether BHB reversed CRC-Oxa resistance via H3K79 demethylation and/or H3K27 deacetylation in vivo and in vitro.

**Results:**

Following BHB intervention based on Oxa, the proliferation, migration, invasion, and EMT of CRC-Oxa cells and the growth and metastasis of transplanted tumors in mice were suppressed. Clinical analysis revealed that the differential change in BHB level was associated with drug resistance and was decreased in drug-resistant patient serum. The H3K79me, H3K27ac, and H3K14ac expressions in CRC were negatively correlated with BHB. Furthermore, results indicated that H3K79me inhibition may lead to BHB target deletion, resulting in its inability to function.

**Conclusions:**

β-hydroxybutyrate resensitized CRC cells to Oxa by suppressing H3K79 methylation in vitro and in vivo.

**Supplementary Information:**

The online version contains supplementary material available at 10.1186/s10020-024-00864-1.

## Background

Colorectal cancer (CRC) is a malignant digestive tract tumor with the highest clinical incidence and mortality rates, which seriously threatens human health and quality of life (Baidoun et al. 2021). In recent years, researchers have developed various chemotherapeutic drugs, such as doxorubicin, gemcitabine, oxaliplatin (Oxa), and so on, for cancer treatment. Among them, Oxa is one of the most commonly used chemotherapeutic drugs (Singh et al. 2022). Currently, the combination chemotherapeutic regimen based on Oxa remains the first-line treatment for CRC, primarily including the FOLFOX regimen (Oxa + calcium folinate + fluorouracil) and the XELOX regimen (capecitabine + Oxa). Oxa can enter cells and cross-link with nucleic acids to produce Pt-DNA adducts to inhibit DNA replication and RNA transcription, thereby promoting the killing of immune and cancer cells by activating the body’s immune response (Guo et al. 2016). In recent years, with continuous improvement of surgical techniques and application of immune and targeted therapies, the treatment for CRC has improved substantially. However, traditional chemotherapeutic and targeted drugs can rapidly develop resistance during the treatment process. According to statistics, approximately 40–50% of patients with stage II and III CRC eventually experience chemotherapy resistance and recurrence during the treatment process (Dariya et al. 2020). Therefore, exploring the Oxa resistance mechanism and overcoming resistance is crucial for the treatment of CRC.

Although genomic changes cause drug resistance, the specific mechanism and mode of drug resistance are still not thoroughly investigated. The occurrence of chemo-resistance is considered permanent and unchangeable. Epigenetics has the characteristic of reversibility; therefore, uncovering drug resistance mechanisms at the epigenetics level may be a potential therapeutic target. Epigenetics refers to the change of gene expression level without DNA sequence changes and has the characteristics of heritability (Li 2021). It most frequently occurs during the cell differentiation process. The current theory believes that malignant tumors occur because of genomic abnormalities and abnormal epigenetic changes, such as DNA methylation, histone modification, and noncoding RNA (Recillas-Targa 2022). These post-translational histone modifications generally include acetylation, methylation, phosphorylation, ubiquitination, SUMOylation, and ADP-ribosylation (Zhang et al. 2021). These modifications usually target amino acid specificity, and the acetylation and methylation modifications of specific lysines of histones H3 and H4 are the most commonly studied. For example, H3K4me3, H3K36me3, and H3K79me3 have transcriptional activation activity, while H3K9me3 and H3K27me3 have transcriptional repression (Zhang et al. 2015; Xiao et al. 2021; Wang et al. 2023). Moreover, histone acetylation increases the repulsive force between histones and DNA by affecting the charge of histones. Therefore, changing the secondary chromatin structure can promote gene transcription (Shvedunova and Akhtar 2022). Various studies have reported that targeted histone modification can participate in tumor progression and improve the drug resistance of tumors. Byun et al. (Byun et al. 2020). reported that aberrant methylation of H3K79me is a potential therapeutic target to elevate the clinical management of triple-negative breast cancer, and H3K79me inhibition can hinder the growth of metastatic triple-negative breast cancer by preventing the progression of epithelial-mesenchymal transition (EMT). Ma et al. (Ma et al. 2022). proposed that PITPNA-AS1 knockdown could sensitize gastric cancer cells to cisplatin treatment by inhibiting H3K27ac. Furthermore, a study confirmed that the high DOT1L and H3K79me2 expression in CRC tissues was a predictor of poor patient survival (Sun et al. 2022). Therefore, histone modification levels may play an indispensable role in many cancers, including CRC. Thus, blocking strategies for histone abnormalities has become a major research hotspot.

Recently, researchers performed dietary screening in animal models of primary cancer and discovered that the ketogenic diet (KD) has a strong tumor suppressive effect (Mundi et al. 2021). KD is a dietary formula that forces body cells to reduce their dependence on carbohydrates, which can use ketone body capacity by adjusting the supply ratio of fat and carbohydrates (Dhamija et al. 2013). In the ketosis state, tumor cells cannot utilize ketone body capacity because of mitochondrial dysfunction. However, this does not affect normal cell ketone body metabolism. Thus, KD minimally impacts normal cell metabolism (Barrea et al. 2022). The abovementioned research shows that KD can inhibit tumor growth, suggesting that it has a good prospect of clinical application. Wamidh et al. (Talib 2020). suggested that KD combined with melatonin could overcome the chemo-resistance of patients with breast cancer, indicating that KD could also aid reduce tumor resistance to chemotherapeutic drugs. As one of the main components of ketone bodies, β-hydroxybutyrate (BHB) can recapitulate these properties of KD. It has been reported that BHB can reduce the proliferation of colonic crypt cells and effectively inhibit intestinal tumor growth (Dmitrieva-Posocco et al. 2022). Furthermore, BHB has histone deacetylase inhibitor properties, which can affect the transcription level of the genome by regulating the histone acetylation modification level and participating in tumor occurrence and development (Xie et al. 2016). However, the effectiveness of BHB on histone modification level and Oxa resistance in CRC still requires further demonstration.

In this study, we used Oxa induction to generate human CRC drug-resistant cell lines and focused on not only the effects of Oxa on drug resistance of drug-resistant cell lines but also whether the underlying mechanism of drug resistance was associated with H3K79 methylation level.

## Methods

### Clinical sample collection

Clinical data of 15 (9 males and 6 females) patients with CRC treated with Oxa-based adjuvant chemotherapy following radical resection in our hospital from April 2020 to September 2022 were collected. Their ages ranged from 36 to 79 years, with four patients aged < 60 years and 11 aged ≥ 60 years. Among them, 13 and 2 patients suffered from stages I–II and stages III–IV CRC, respectively. The primary tumor location was the colon and rectum in 5 and 10 patients, respectively. Eight patients were Oxa-sensitive (responder) and seven were Oxa-resistant (nonresponder). Inclusion criteria: all patients who met the diagnostic criteria for CRC and were confirmed by pathological examination; those who did not receive chemoradiotherapy or immunotherapy preoperatively and with surgical resection specimens archived in paraffin; those indicated for Oxa-based postoperative adjuvant chemotherapy; and those with complete clinical data. Exclusion criteria: patients with other tumors; with acute and chronic enteritis, inflammatory bowel disease, and other digestive system diseases; and with acute urinary infection, tuberculosis infection, and other infectious diseases. The chemosensitivity and drug resistance were assessed based on the evaluation criteria of solid tumor efficacy: all targets and/or residual lesions disappeared and maintained for 4 weeks and were recorded as complete remission. The total length and diameter of the baseline target and/or residual lesions were reduced by 30% and maintained for 4 weeks and recorded as partial remission. If the total length and diameter of the baseline target and/or residual lesions were reduced but did not meet the criteria for partial remission, they were recorded as stable. An increase of ≥ 20% in the total length and diameter of the baseline target and/or residual lesions was recorded as progression. Complete remission and partial remission were recorded as chemotherapy-sensitive, and stability and progression were recorded as chemotherapy-resistant. All extractions were reviewed and approved by the medical ethics committee of Jiamusi University with informed patient consent.

### Oxa-resistant strain establishment

The human CRC cell lines HCT116 (Sunncell, Wuhan, China) and LoVo (Procell, Wuhan, China) were cultured in Dulbecco’s Modified Eagle Medium (DMEM) containing 10% fetal bovine serum (FBS, Thermo Fisher, USA) in a 5% CO_2_-saturated humidified cell incubator at 37 °C. Oxa-resistant cell lines were established using the drug concentration escalation approach. In short, HCT116 and LoVo cells in the logarithmic growth phase (60–80% confluence) were taken and gradually stimulated with 0.1, 1, 3, 5, 7, and 10 µM Oxa (Sigma-Aldrich, USA) in the culture medium. After continuous cultivation for 4 weeks, each concentration was replaced with the next higher concentration until 10 µM Oxa was added for 4 weeks. The cell lines passed three times at each drug concentration, and the cell bottles were frozen at each increase in drug concentration. Repeated testing was conducted on all cell lines to determine that they do not contain *Mycoplasma* (*Mycoplasma* PCR detection kit, Minerva Biolabs, Berlin, Germany). Then, cells were collected to evaluate the IC50 of Oxa on HCT116 and LoVo parental and drug-resistant cells using the CCK-8 assay.

### Cell processing and grouping

Based on the experimental design, HCT116-Oxa and LoVo-Oxa cells were divided into four groups: control, Oxa, BHB, and Oxa + BHB. For the control group, cells were cultured normally without any treatment. For the Oxa group, 10 µM of Oxa was added to the culture medium to incubate the cells for 48 h. For the BHB group, 4 mM of BHB was added to the culture medium to incubate the cells for 48 h. For the Oxa + BHB group, 10 µM of Oxa and 4 mM of BHB were coadded in the medium to incubate the cells for 48 h. Furthermore, HCT116-Oxa cells were divided into six groups: Oxa, Oxa + BHB, Oxa + sh-DOT1L, Oxa + sh-DOT1L + BHB, Oxa + GNE-049, and Oxa + GNE-049 + BHB. For the Oxa + sh-DOT1L group, HCT116-Oxa cells were transfected with sh-DOT1L for 48 h and then treated with 10 µM of Oxa. For the Oxa + sh-DOT1L + BHB group, HCT116-Oxa cells were transfected with sh-DOT1L for 48 h and then treated with 10 µM of Oxa and 4 mM of BHB. For the Oxa + GNE-049 group, 10 µM of Oxa and 10 µM of GNE-049 (MedChemExpress, NJ, USA) were simultaneously added to the medium to incubate the cells for 48 h. For the Oxa + GNE-049 + BHB group, 10 µM of Oxa, 10 µM of GNE-049, and 4 mM of BHB were simultaneously added to the medium to incubate the cells for 48 h.

### Cell transfection

The shRNAs against DOT1L were designed by Qiagen (Qiagen, Germany). To avoid off-target effects, three different shRNA constructs were designed (sh-DOT1L#1, #2, and #3). Furthermore, when preparing dilutions of the transfection reagent, DMEM without serum but with antibiotics was used. This was because the serum can affect the complex formation whereas antibiotics are generally nontoxic to eukaryotic cells (thereby avoiding cell staining). Confluent cells were diluted in DMEM medium. Cell growth was observed till approximately 70% confluency. This was when the cell monolayer was covered with DMEM without serum; however, with antibiotics, plasmid transfection was performed using Lipofectamine®3000 transfection reagent and as required for experiments.

To establish a stable transfection cell line, sh-DOT1L#2 was transfected into HCT116-Oxa cells. At 10 days post-transfection, puromycin-resistant cell pools were selected.

### Animal experiments

Animal experiments were approved and supervised by the Animal Ethics Committee of Jiamusi University. In total, seventy-two 5-week-old male athymic BALB/C nude mice were obtained from the Guangdong Experimental Animal Center. Among these, 32 were selected to construct a CRC subcutaneous tumor model. They were randomly divided into fourgroups (each group comprised six mice): control (Saline), Oxa (5 mg/kg/day), BHB (40 mg/mice/day), and Oxa + BHB. The 8 × 10^6^ HCT116-Oxa cells were suspended in 0.2 mL phosphate-buffered saline and subcutaneously inoculated into the peritoneum of the mice. Subsequently, 5 mg/kg Oxa or 40 mg/mice BHB were intraperitoneally injected daily based on the group. Three mice of each group were sacrificed to collect tumors for detecting cleaved-caspase 3 on the 15th of drug intervention The tumor volume of the remaining mice was measured every 5 days for 1 month of continuous treatment. The remaining mice were sacrificed on the 30th day of drug intervention, and tumors were collected for weight and the protein expression analysis.

Furthermore, 20 nude mice were selected to construct the CRC metastasis model simultaneously. They were grouped in a manner that was same as mentioned above. A mouse model of liver metastasis from CRC was established by intrasplenic implantation. Anesthetized by intraperitoneal injection of 1% pentobarbital sodium at 45 mg/kg, a 0.5–1.0 cm oblique incision was made on the left back (below the junction of the left posterior axillary line and the costal margin). After exposing the spleen in the abdomen, the lower part of the spleen was gently lifted out of the abdominal cavity and 8 × 10^6^ HCT116-Oxa cells were slowly injected into the spleen of nude mice using a needle. Each mouse was injected with 0.2 ml of cell suspension for approximately 3 min. During this period, the splenic capsule swelled and turned white. After the injection was completed and the needle was pulled out, the needle eye was compressed with a 75% ethanol cotton swab for 2 min to compress hemostasis and kill cancer cells that may extravasate to prevent intraperitoneal implantation and metastasis. Eventually, the spleens of nude mice were put back in situ and the abdomen was closed. The animals were raised routinely after awakening from anesthesia. The whole operation process followed the principle of aseptic operation. The mice were sacrificed on the 30th day of drug intervention, and liver tissues were removed for HE staining and the number of metastatic nodules was observed.

Subsequently, based on the experimental design, 20 nude mice were selected to construct the transplanted tumor model, which was divided into four groups: Oxa, Oxa + BHB, sh-DOT1L + Oxa, and sh-DOT1L + Oxa + BH. For the sh-DOT1L + Oxa and sh-DOT1L + Oxa + BHB groups; each group comprised six mice. Then, 8 × 10^6^ HCT116-Oxa cells stably transfected with sh-DOT1L were suspended in 0.2 mL phosphate-buffered saline and subcutaneously inoculated into the mouse peritoneum. The subsequent treatments were the same as those mentioned above.

### Cell viability assay

HCT116-Oxa and LoVo-Oxa cells after different treatment groups were made into single-cell suspensions and seeded in 96 well plates (2000 cells/well), respectively, with three replicate wells set for each time point. A 10 µL of CCK-8 reagent (Sigma-Aldrich, USA) was added to the wells to be examined at 24 h, and incubation was continued at 37 °C for 3 h. The absorbance at 450 nm was determined in a microplate reader (Bio-Rad, USA). GraphPad Prism 7.0 software was used to draw graphs and histograms of HCT116 and LoVo cell viability, and IC50 curves of drugs were plotted.

### Clone formation assay

HCT116-Oxa and LoVo-Oxa cells from each group with different treatments were collected and trypsinized. Cell suspensions were seeded in 6-cm-diameter dishes and incubated in a saturated humidity, 37 °C, 5% CO_2_ incubator for 21 consecutive days at 200 cells per dish. Subsequently, 5 mL of methanol (Shanghai Yuanye Biotechnology Co., Ltd, China) was used to fix the cells for 15 min, and Giemsa staining solution (Sigma-Aldrich, USA) was used to stain the cells for 20 min. The number of cell colonies was counted under a 100× microscope, where the criterion for being considered a colony was that the cell mass containing > 50 cells was one cell colony.

### Cell apoptosis assay

HCT116-Oxa and LoVo-Oxa cells from each group after transfection were plated at a density of 1 × 10^4^ cells/well seeded in 24 well plates and cultured for 48 h. After the cells were collected, cell apoptosis was checked by flow cytometry (Attune NxT, ThermoFisher, USA) following the instructions of Annexin V-FITC/PI kit (Pricella, Wuhan, China).

### Cell invasion and migration assays

Matrigel was added into the Transwell chamber, and the 24 well plate containing the chamber was placed in the cell incubator to accelerate Matrigel solidification. HCT116-Oxa and LoVo-Oxa cell suspensions containing 2.5 × 10^4^ different treatments were added to the upper chamber, and 500 µL of DMEM was added to the lower chamber. After 24 h, the medium was removed, and the cells were incubated with paraformaldehyde and crystal violet. Finally, invasive cells were observed and counted under a microscope. For cell migration detection, the other steps are the same as above except that Matrigel is not added.

### Western blotting

Cell lysates were used to extract total cellular protein after different treatments. The proteins on the gels were transferred to nitrocellulose membranes by sodium dodecyl-sulfate polyacrylamide gel electrophoresis. Nitrocellulose membranes were blocked with Tris-buffered saline with 0.1% Tween® 20 detergent containing 5% nonfat dry milk for 2 h. Subsequently, primary antibodies (E-cadherin, N-cadherin, Vimentin, Bax, Bcl-2, DOTIL, H3K79me, and H3K27ac) were added and incubated overnight at 4 °C with shaking. Secondary antibodies of goat anti-rabbit IgG were then added for incubation at room temperature for 2 h. ECL luminescent solution was added to the nitrocellulose membrane to react for 1–3 min and exposed in a dark room. The relative expression levels of proteins were analyzed using Image pro-plus 5.0 software, and GAPDH was used as a loading control. The antibodies against Bax (#AF1270), Bcl-2 (#AF0060), E-cadherin (#AF6759), N-cadherin (#AF0243), Vimentin (#AF1975), and DOTIL (#AF6720) were purchased from Beyotime, whereas those against H3K79me (#ab177184) and H3K27ac (#ab177178) were provided by Abcam.

### Immunohistochemical analysis

The cancer tissues left after surgical resection were collected. All specimens were fixed with 10% neutral formaldehyde, then embedded in paraffin, and made into 4 μm thick sections. Antigen retrieval was performed with 0.01 mol/L citrate buffer, followed by immunohistochemical staining and light microscopic observation. Cancer tissue specimens and adjacent tissue specimens were verified by immunohistochemistry (IHC) after being reviewed and confirmed by three senior pathologists. The specimens were assessed according to the positive percentage of tumor-pigmented cells. Information of antibodies for IHC was as follows: Ki-67 (#MA5-14520; Thermofisher), cleaved-caspase 3 (#25128-1-AP; ProteinTech), H3K79me (#ab177184; Abcam), H3K27ac (#ab177178; Abcam), H3K14ac (#MA5-32814; Thermofisher), and H3K9me (#ab8898; Abcam).

### HE staining

The liver tissues of nude mice in each group were excised and fixed with 4% paraformaldehyde at 4 °C for a week. After tissue paraffin embedding and paraffin block sectioning, hematoxylin, and eosin staining (Thermofisher, USA) were used based on the manufacturer’s requirements. Finally, the slides were blocked after dehydration with ethanol, and then, the staining was observed under a microscope.

### RT-qPCR

HCT116-Oxa and LoVo-Oxa cells from each group with different treatments were collected, and the total RNA was extracted. The cDNA was then used as a template for DOT1L amplification on a Bio-Rad CFX90 real-time PCR. RT-PCR reaction conditions: predenaturation at 95 °C for 30 s, 39 cycles of denaturation at 95 °C for 5 s, annealing at 60 °C for 5 s, and extension at 65 °C for 5 s. The primers are listed in Supplementary Table S1. GAPDH was used as an internal reference, and the relative expression levels were calculated using the 2^−ΔΔCT^ method.

### Statistical analysis

Experimental data were statistically analyzed using GraphPad Prism 7.0 software. All experiments were triplicate independent experiments, and one-way analysis of variance was used to compare three groups and above, and a t-test was used between two groups, with *P* < 0.05 indicating a statistical difference.

## Results

### BHB reversed the acquired resistance of CRC cells to Oxa

**Fig. 1:**
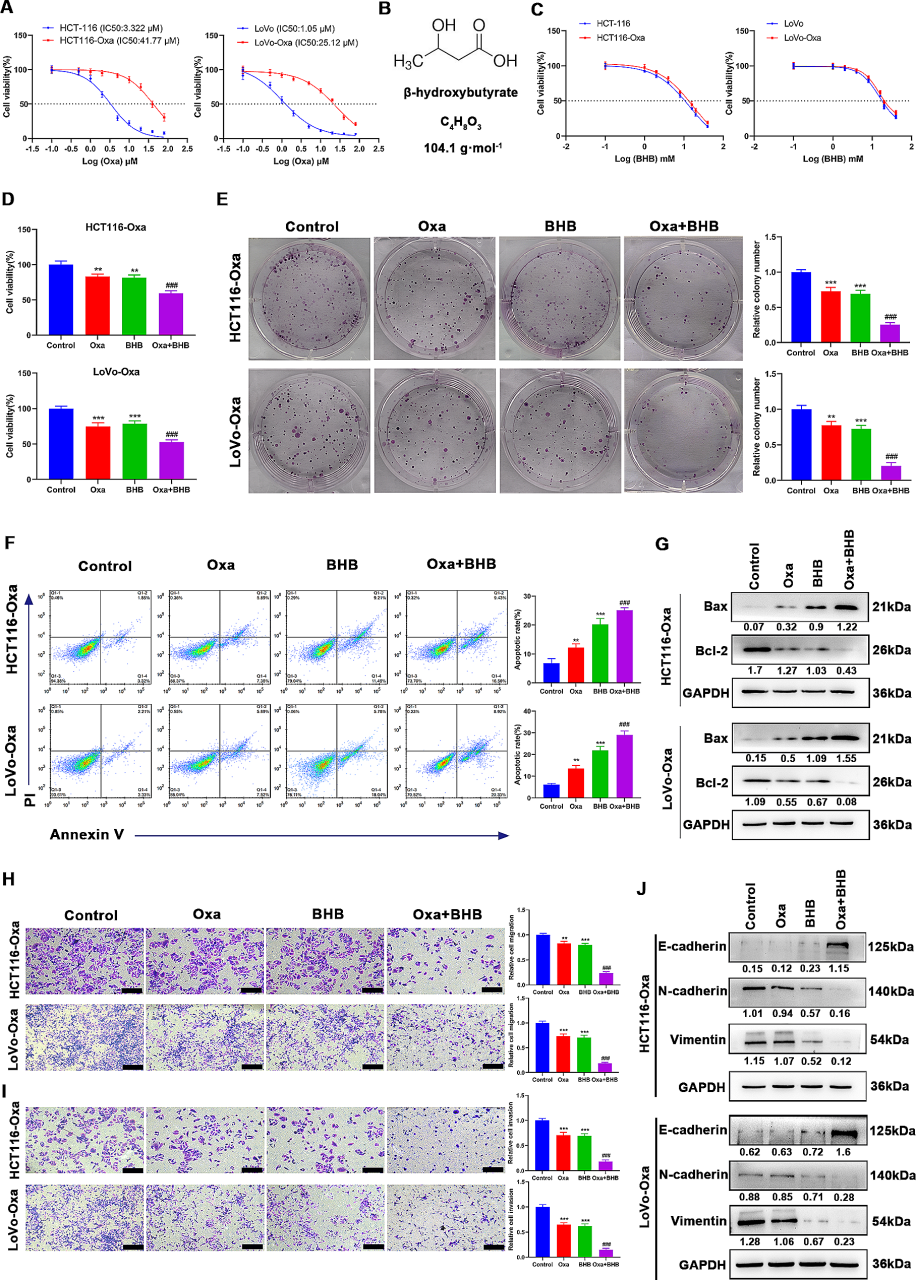
BHB reversed the acquired resistance of CRC cells to Oxa. **A**: CCK-8 determination of the cell viability of HCT-116 and LoVo parental and drug-resistant cells treated with Oxa at a series of concentrations (0, 0.1, 0.5, 1, 2, 5, 10, 20, 40, and 80 µM) for 24 h, and then, the IC50 value was calculated. **B**: Chemical structural formula of BHB. **C**: CCK-8 determination of the cell viability of HCT-116 and LoVo parental and drug-resistant cells treated with BHB at a series of concentrations (0, 1, 2, 4, 8, 16, 20, and 40 mM) for 24 h. **D**: CCK-8 determination of the cell viability of HCT-116-Oxa and LoVo-Oxa cells incubated with Oxa (10 µM) and/or BHB (4 mM) for 24 h. **E**: Clone formation determination of the cell proliferation of HCT-116-Oxa and LoVo-Oxa cells incubated with Oxa (10 µM) and/or BHB (4 mM) for 24 h. **F**: Annexin V-FITC/PI double staining determination of cell apoptosis of HCT-116-Oxa and LoVo-Oxa cells incubated with Oxa (10 µM) and/or BHB (4 mM) for 24 h. **G**: Western blotting determination of Bax and Bcl-2 protein levels in HCT-116-Oxa and LoVo-Oxa cells incubated with Oxa (10 µM) and/or BHB (4 mM) for 24 h. **H–I**: Transwell assay determination of the invasion and migration ability of HCT-116-Oxa and LoVo-Oxa cells incubated with Oxa (10 µM) and/or BHB (4 mM) for 24 h. **J**: Western blotting determination of E-cadherin, N-cadherin, and vimentin protein levels in HCT-116-Oxa and LoVo-Oxa cells incubated with Oxa (10 µM) and/or BHB (4 mM) for 24 h. Data: Mean ± SEM. **P < 0.01, ***P < 0.001 vs. Control group. ###*P* < 0.001 vs. Oxa group (*n* = 3)

First, HCT116-Oxa- and LoVo-Oxa-resistant strains were established by exposing HCT116 and LoVo cells to Oxa with increasing concentrations (0.1–10 µM), wherein the establishment cycle was 10 months. After the CRC-Oxa-resistant strain was successfully established, a series of concentrations (0, 0.1, 0.5, 1, 2, 5, 10, 20, 40, and 80 µM) of Oxa was used to treat resistant strains and parental strain cells for 24 h. Figure 1A reveals that the IC50 of the HCT116-Oxa-resistant strain was 41.77 µM and that of LoVo-Oxa-resistant strain was 25.12 µM. Next, the cells were treated with BHB at different concentrations (0, 1, 2, 4, 8, 16, 20, and 40 mM) for 24 h to show the effects of BHB on the viability of CRC parental strains and drug-resistant strains. Figure 1B shows the BHB chemical structure formula. Figure 1C showed that BHB suppressed the viability of HCT116 and LoVo parental strains and drug-resistant strains in a dose-dependent manner. Moreover, the response of sensitive and drug-resistant cell lines to BHB was similar. Subsequently, HCT116-Oxa and LoVo-Oxa cells were cultured with 10 µM of Oxa and 4 mM of BHB alone or together. The results of the cell viability and proliferation test displayed that Oxa and BHB alone could restrain the viability and proliferation of drug-resistant cells, while the viability and proliferation of drug-resistant cell lines in the Oxa + BHB co-treatment group were conspicuously lower than those in the Oxa-alone group (Fig. 1D and E). These data suggested that the sensitivity of CRC drug-resistant cell lines to Oxa was enhanced after the BHB intervention. In addition, both the treatment with Oxa and BHB could lead to the apoptosis of Oxa-resistant CRC cells, while their combination exerted a synergism (Fig. 1F and G). The results of cell invasion and migration assays indicated that the number of invasive and migrated cells and the N-cadherin and vimentin protein expressions were unusually reduced compared with that without BHB treatment, while the protein E-cadherin level was upregulated (Fig. 1H and J). These results further supported that the sensitization effect of Oxa on CRC drug-resistant cell lines was enhanced after the BHB treatment, In conclusion, BHB can reverse the acquired resistance of CRC-resistant strains to Oxa.

### BHB resensitized CRC to Oxa in vivo

**Fig. 2:**
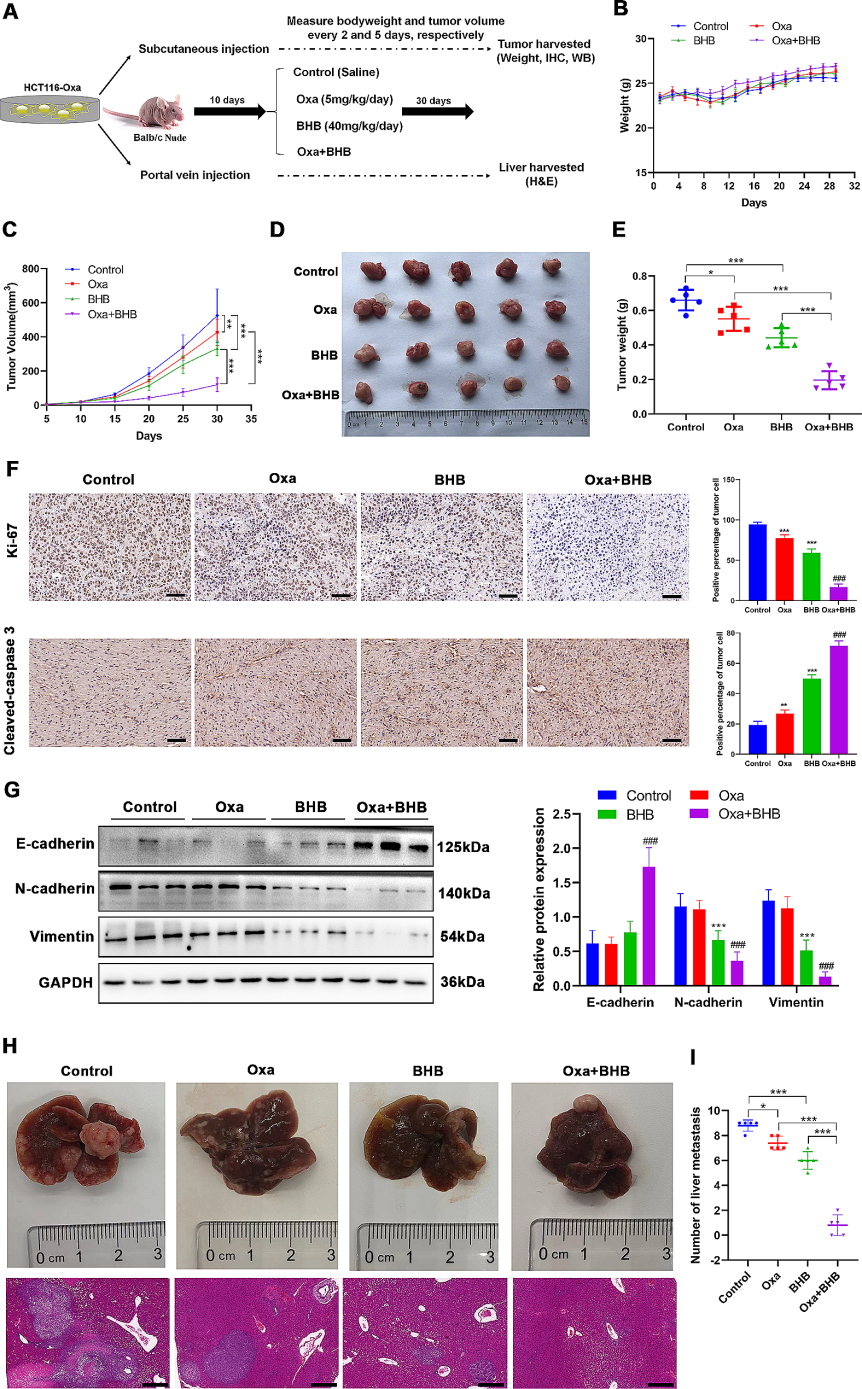
BHB resensitized CRC to Oxa *in vivo.***A**: The animal experiment process. The tumor-bearing nude mice were randomly divided into four groups (*n* = 6): control (saline), Oxa (5 mg/kg/day), BHB (40 mg/mice/day), and Oxa + BHB. **B**: From the first day of administration, the weight of nude mice was measured once every 2 days. **C**: The tumor growth curve of each group. **D**: Representative diagram of tumors in each group. **E**: The tumor weight of each group. **F**: Immunohistochemical determination of the Ki67 (day 30) and cleaved-caspase3 (day 15) expressions in tumor tissues of subcutaneous tumor model mice after injecting Oxa (5 mg/kg/day) and/or BHB (40 mg/mice/day), and the immunohistochemical score was calculated. **G**: Western blotting determination of the E-cadherin, N-cadherin, and vimentin expression levels in tumor tissues of subcutaneous tumor model mice after injecting Oxa (5 mg/kg/day) and/or BHB (40 mg/mice/day). **H**: Liver metastasis histograms and HE staining sections of nude mice in each group. **I**: Statistics of liver metastatic nodules of nude mice in each group. Data: Mean ± SEM. *P < 0.05, ***P < 0.001 vs. Control group. ###*P*<0.001 vs. Oxa group (*n* = 6)

Next, we further evaluated whether BHB has the same effect in animal experiments. First, the CRC subcutaneous tumor model and metastasis model were established by subcutaneous injection or a portal vein injection of 8 × 10^6^ HCT116 Oxa cells into mice, and then injecting Oxa (5 mg/kg/day) and/or BHB (40 mg/mice/day) every day for 1 month (Fig. 2A). The body weight of mice in each group was measured every 2 days from the first day of administration. The results displayed that the body weight of mice in each group showed an increasing trend, without significant difference (Fig. 2B). However, the tumor volume and weight in the Oxa + BHB group were materially lower than in the Oxa-alone treatment group (Fig. 2C and E). Subsequently, to measure the proliferation and apoptosis of tumor cells in vivo, the Ki67 and cleaved-caspase3 expressions in tumor tissues of subcutaneous tumor model mice were evaluated at days 30 and 15, respectively, by IHC. The immunohistochemical results demonstrated that Ki67 expression in tumor tissues of the Oxa + BHB group was prominently lower than that of the Oxa alone, whereas cleaved-caspase3 expression was significantly upregulated (Fig. 2F), indicating that consistent with the results of in vitro experiments, BHB intervention enhanced the sensitivity of CRC cells to Oxa. The detection results of EMT also confirmed that compared to the Oxa or BHB alone treatment group, the Oxa + BHB cotreatment group showed an upregulation of E-cadherin protein levels and a significant decrease in N-cadherin and vimentin protein expression (Fig. 2G). Next, the analysis of liver tissue of metastasis model mice revealed that the number of liver metastasis nodules in the Oxa treatment group decreased from an average of nine in the control group to an average of seven, while the average number of liver metastasis nodules in the Oxa + BHB cotreatment group was only one (Fig. 2H and I). In conclusion, BHB can resensitize CRC to Oxa in vivo, regardless of the subcutaneous tumor model or metastasis model.

### Serum BHB level is associated with oxa resistance in patients with CRC

**Fig. 3:**
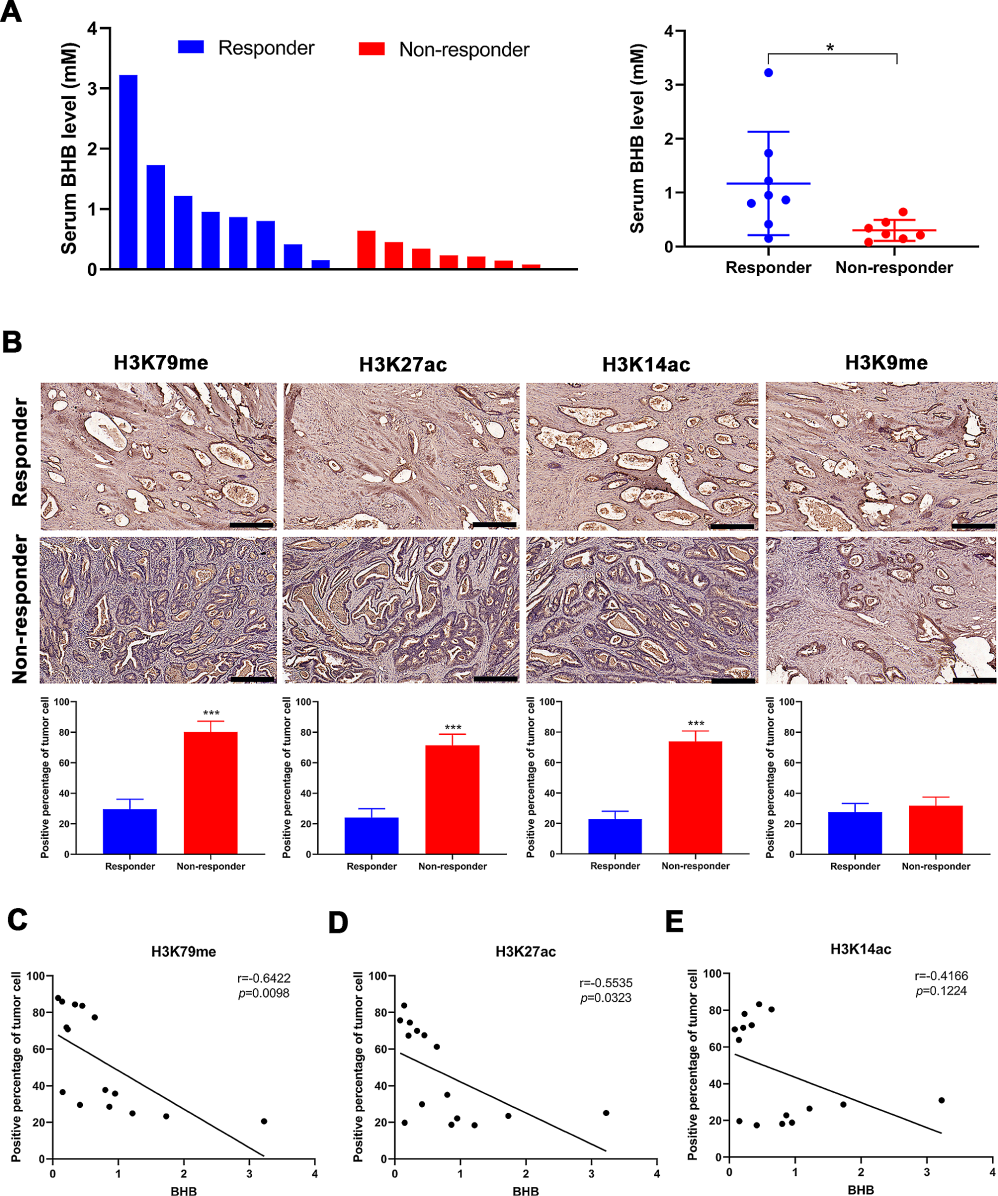
Serum BHB level is associated with Oxa resistance in patients with CRC. **A**: The serum of eight Oxa-sensitive and seven drug-resistant patients was collected after Oxa chemotherapy, and the BHB content was analyzed by β-hydroxybutyrate (ketone body) colorimetric assay kit. **B**: Immunohistochemical determination of the H3K79me, H3K27ac, H3K14ac, and H3K9me expressions in tumor tissues of 15 patients with CRC, including eight Oxa-sensitive (responder) and seven nonresponder patients. **C**: Correlation analysis between H3K79me tissue score and serum BHB level. **D**: Correlation analysis between H3K27ac tissue score and serum BHB level. **E**: Correlation analysis between the H3K14ac tissue score and serum BHB level. Data: Mean ± SEM. *P < 0.05, ***P < 0.001 vs. the responder group

Subsequently, serum samples were collected from 15 patients with CRC on similar regular diets and without KD, including eight Oxa-sensitive (responder) and seven nonresponder patients, to detect BHB levels. Taking the median level as the boundary, patients were divided into two groups with high and low BHB levels to analyze the correlation between BHB and clinical characteristics of patients (gender, age, tumor stage, tumor location, and drug resistance). The results revealed that differential changes in BHB expression levels were only associated with drug resistance (Table 1). Figure 3A showed that the BHB levels in the serum of Oxa-resistant patients were significantly lower than those of Oxa-sensitive patients. Next, IHC was performed to check the expression levels of H3K79me, H3K27ac, H3K14ac, and H3K9me in the tumor tissues of 15 patients with CRC. The computed immunohistochemical score suggested that H3K79me, H3K27ac, and H3K14ac were prominently overexpressed in tumor tissues of drug-resistant patients, while the H3K9me expression levels in drug-resistant patients were consistent with those in Oxa-sensitive patients (Fig. 3B). The in vitro analysis further revealed that the H3K79 methylation and H3K27 acetylation levels were higher in HCT116 and LoVo-resistant cell lines (Figure S1A). Moreover, HCT116-Oxa and LoVo-Oxa cells were treated with a series of concentrations (0, 1, 2.5, 5, and 10 µM) of BHB for 24 h, and the H3K79 methylation and H3K27 acetylation levels were measured by Western blotting to explore whether BHB plays a regulatory role on H3K79 methylation and H3K27 acetylation. Figure S1B shows that BHB intervention downregulated H3K79 methylation and H3K27 acetylation levels in a dose-dependent manner in HCT116-Oxa and LoVo-Oxa cells (Figure S1B). Next, the correlation between BHB level and H3K79me, H3K27ac, and H3K14ac positive cell ratio in tumor tissues of 15 patients with CRC was comprehensively analyzed. It was concluded that H3K79me and H3K27ac expressions in CRC were significantly negatively correlated with serum BHB levels (Fig. 3C–E). Moreover, in vitro analysis indicated that Oxa couldn’t affect the H3K79me and H3K27ac levels in HCT116-Oxa and LoVo-Oxa cells. However, both H3K79me and H3K27ac levels were decreased by the combined treatment of Oxa + BHB, compared with the Oxa-alone treatment (Figure S1C–S1D). These results suggested that H3K79me and H3K27ac levels correspond to the Oxa sensitivity with combined treatment.

**Table 1:** Association between BHB level and clinical characteristics

		All patients (*n*)	High level (*n*)	Low level (*n*)	*P*-value
Total number		15	7	8	
Gender	Male	9	4	5	1.000
	Female	6	3	3	
Age	< 60	4	1	3	0.571
	≥ 60	11	6	5	
Tumor grade	I–II	13	5	8	0.200
	III–IV	2	2	0	
Primary tumor site	Colon	5	3	2	0.608
	Rectum	10	4	6	
Oxaliplatin response	Responder	8	6	2	0.041
	Non-responder	7	1	6	

### Resensitizing CRC cells to oxa by BHB mainly relied on H3K79 methylation suppression

**Fig. 4:**
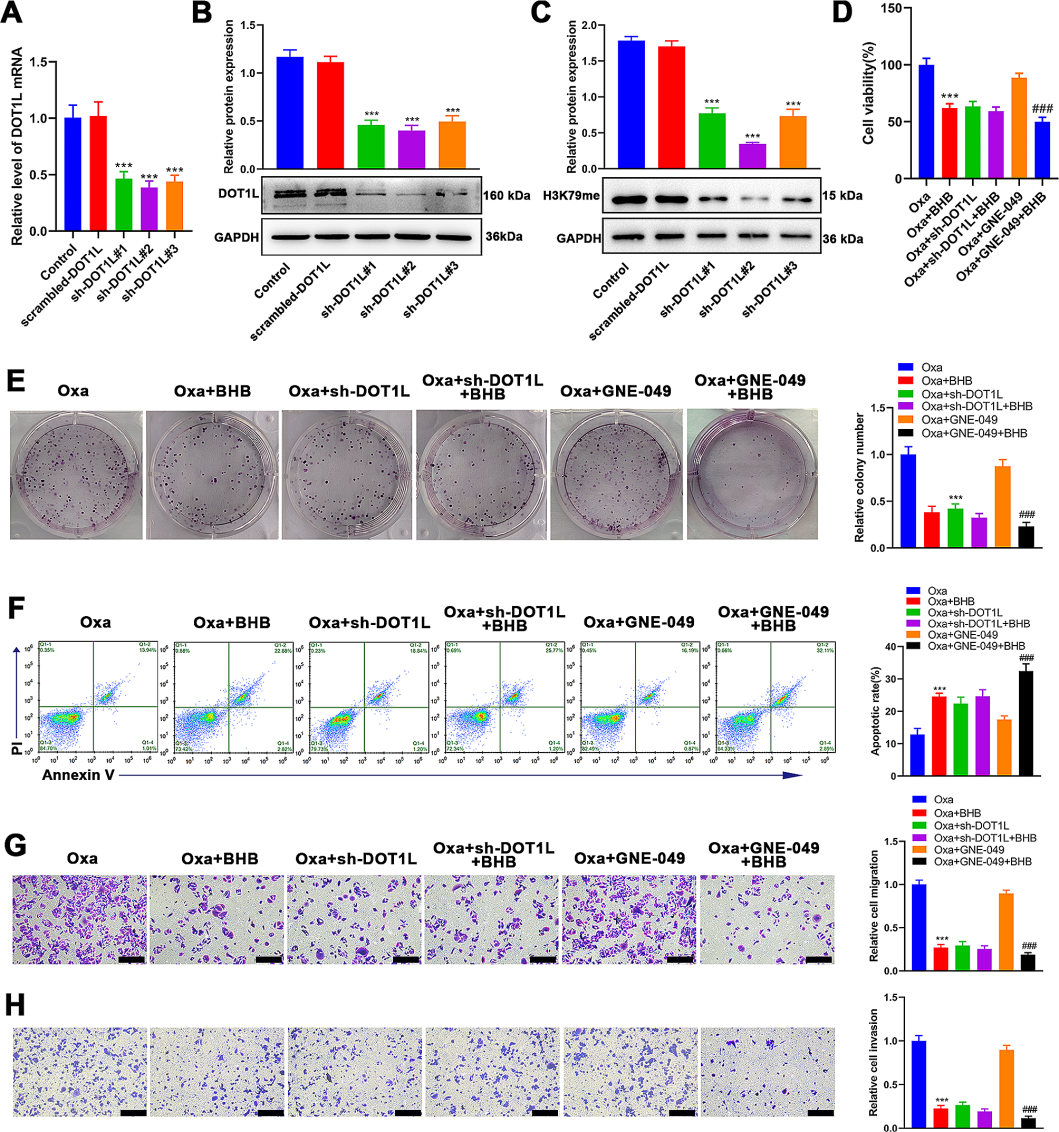
Resensitizing CRC cells to Oxa by BHB mainly relied on H3K79 methylation suppression. **A**: RT-qPCR determination of the mRNA level of DOT1L in HCT-116-Oxa cells, which transfected with scrambled-DOT1L, sh-DOT1L#1, sh-DOT1L#2, and sh-DOT1L#3. **B**: Western blotting determination of the protein level of DOT1L in HCT-116-Oxa cells, which transfected with scrambled-DOT1L, sh-DOT1L#1, sh-DOT1L#2, and sh-DOT1L#3. **C**: CCK-8 determination of the cell viability of HCT-116-Oxa cells, which inhibited H3K79 methylation or H3K27 acetylation and/or BHB intervention. **D**: Clone formation determination of the cell proliferation of HCT-116-Oxa cells, which inhibited H3K79 methylation or H3K27 acetylation and/or BHB intervention. **E**: Annexin V-FITC/PI double staining determination of the cell apoptosis of HCT-116-Oxa cells, which inhibited H3K79 methylation, H3K27 acetylation, and/or BHB intervention. **F**: Transwell assay determination of the cell migration of HCT-116-Oxa cells, which inhibited H3K79 methylation, H3K27 acetylation, and/or BHB intervention. **G**: Transwell assay determination of the cell invasion of HCT-116-Oxa cells, which inhibited H3K79 methylation, H3K27 acetylation, and/or BHB intervention. Data: Mean ± SEM. ***P < 0.001 vs. Oxa group. ###*P* < 0.001 vs. Oxa + GNE-409 group (*n* = 3)

The clinical analysis results of this study revealed that BHB levels in patients with CRC were negatively correlated with H3K79 methylation and H3K27 acetylation. Further, previous studies have reported that BHB could suppress H3K79 methylation and also had strong deacetylase activity. Therefore, next, we explored whether BHB reversed the CRC-Oxa resistance via H3K79 demethylation and/or H3K27 deacetylation. In this part, HCT116-Oxa cells were used to restrain H3K79 methylation by knocking down DOT1L, a H3K79 methyltransferase, and H3K27 acetylation using GNE-049, a H3K27 acetylation inhibitor. First, we constructed three DOT1L knockdown vectors, sh-DOT1L#1, sh-DOT1L#2, and sh-DOT1L#3, and then transfected them into HCT116-Oxa cells, respectively. Combining the results of Western blotting and RT-qPCR, the knockdown efficiency of sh-DOT1L#2 was the highest (Fig. 4A and B). Furthermore, Western blotting confirmed that DOT1L knockdown successfully caused a direct decrease in H3K79 methylation (Fig. 4C). After the BHB treatment based on the knockdown of DOT1L, no significant changes in the cell activity (Fig. 4D), proliferation ability (Fig. 4E), apoptosis rate (Fig. 4F), apoptotic protein level (Figure S2A), migration and invasion ability (Fig. 4G and H), and EMT protein expression level (Figure S2B) of HCT116-Oxa cells. It is suggested that the inhibition of H3K79 methylation may lead to the lack of a BHB target, resulting in its inability to play a role in reversing Oxa resistance. After restraining H3K27 acetylation with GNE-049, the cell viability, proliferation, apoptosis, migration, invasion, and EMT of HCT116-Oxa cells had no significant changes (Fig. 4D–H). Moreover, based on the GNE-049 treatment, HCT116-Oxa cell viability (Fig. 4D), proliferation ability (Fig. 4E), migration and invasion ability (Fig. 4G and H), apoptosis inhibitory protein Bcl-2 expression (Figure S2A), and EMT-related protein N-cadherin and vimentin expression (Figure S2B) were pre-eminently reduced, while the cell apoptosis rate (Fig. 4F), apoptosis marker protein Bax level (Figure S2A), and EMT-related protein E-cadherin expression (Figure S2B) were particularly increased by BHB. It suggested that the role of BHB in reversing Oxa resistance was independent of H3K27 acetylation. Therefore, the resensitization of CRC cells to Oxa by BHB primarily depends on the inhibition of H3K79 methylation.

### BHB reversed Oxa resistance by suppressing H3K79 methylation in vivo

**Fig. 5:**
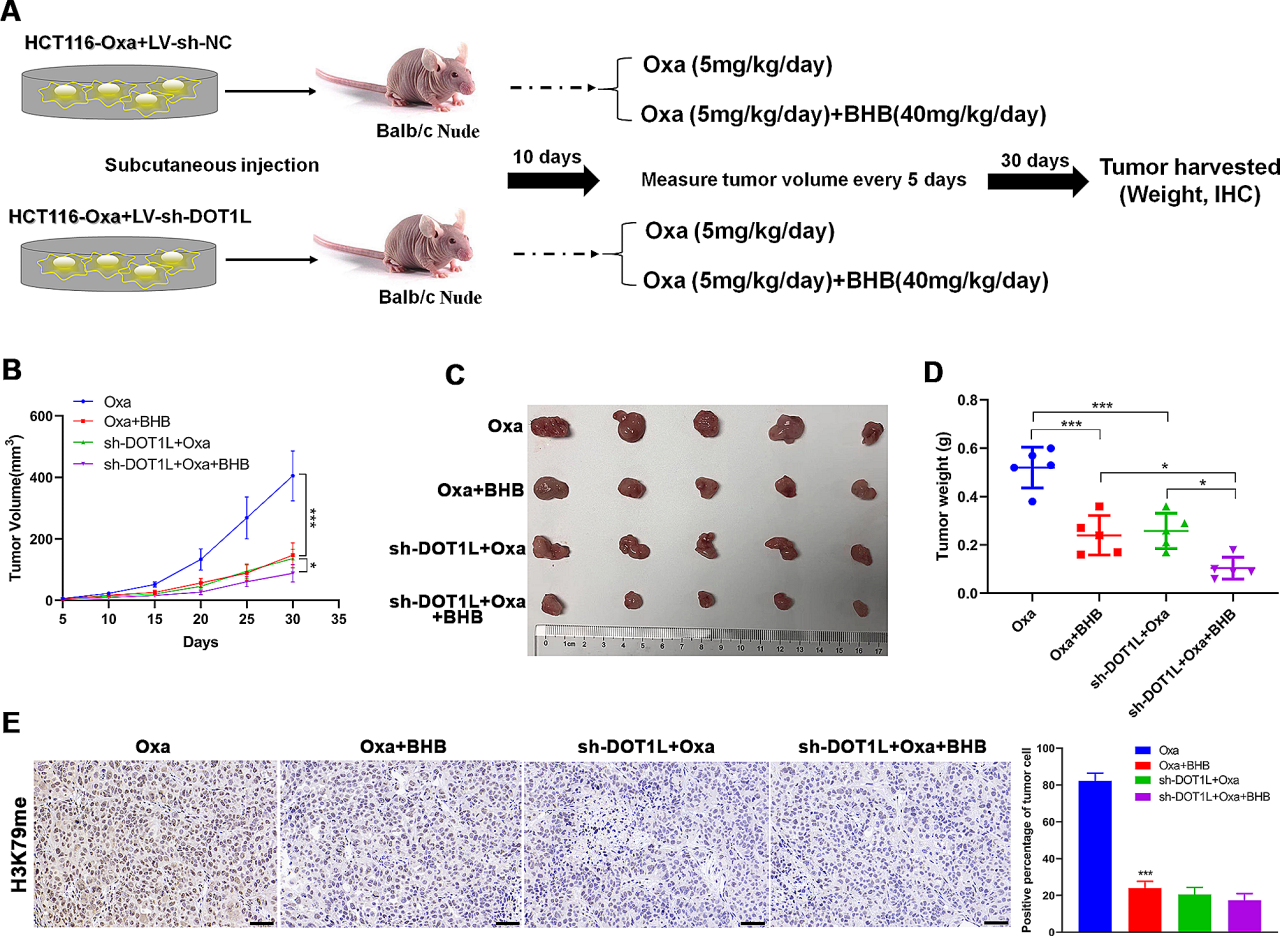
BHB reversed Oxa resistance by suppressing H3K79 methylation in vivo. **A**: The animal experiment process. The tumor-bearing nude mice were randomly divided into four groups (*n* = 6): Oxa (5 mg/kg/day), Oxa + BHB (40 mg/mice/day), sh-DOT1L + Oxa, and sh-DOT1L + Oxa + BHB. **B**: The tumor growth curve of each group. **C**: Representative diagram of tumors in each group. **D**: The tumor weight of each group. **E**: Immunohistochemical determination of the expression of H3K79me in tumor tissues of subcutaneous tumor model mice, and the immunohistochemical score was calculated. Data: Mean ± SEM. ***P < 0.001 vs. Oxa group (*n* = 6)

Next, the subcutaneous transplantation tumor model was constructed to further confirm whether the knockdown of DOT1L could counteract BHB treatment in vivo experiments. Figure 5A shows the flow chart of animal experiments. Following 1 month of continuous treatment, the size (Fig. 5C), volume (Fig. 5B), and weight (Fig. 5D) of transplanted tumors in DOT1L knockdown mice were found to be noticeably reduced by measuring the tumor volume and weight. The tumors further reduced in size and volume after the BHB treatment. Interestingly, no significant changes were observed in H3K79 methylation level after the BHB injection based on DOT1L knockdown, which further confirmed the possibility that H3K79me was regarded as a target by BHB (Fig. 5E). The above findings suggest that BHB reversed the Oxa resistance by inhibiting H3K79 methylation in vivo.

## Discussion

The number of deaths caused by CRC ranks second in the total number of cancer deaths worldwide, causing > 850,000 deaths every year. Epidemiological survey shows that the incidence and mortality of CRC are increasing year by year among people aged < 50 years (Jin et al. 2020). At present, surgery combined with chemoradiotherapy is one of the main treatments for CRC. Studies have found that CRC will have a good therapeutic effect in the early stage of chemotherapy; however, drug resistance may occur in the late stage. The overall survival of patients with advanced CRC is < 2 years even with standardized chemotherapy (Jeught et al. 2018). Oxa, as the key drug of CRC chemotherapy, can prolong the survival of patients to a certain extent. However, Oxa can easily become resistant, and its complex resistance mechanism has not been fully elucidated so far. Therefore, exploring the mechanism of Oxa resistance is of great significance for the prediction of therapeutic efficacy and overcoming tumor resistance. In this study, HCT116-Oxa- and LoVo-Oxa-resistant strains were established by exposing HCT116 and LoVo cells to Oxa at increasing concentrations (0.1–10 µM). Subsequently, the IC50 of the HCT11-Oxa-resistant strain was 41.77 m, and that of the LoVo-Oxa-resistant strain was 25.12 µM, suggesting that the drug-resistant cell line was successfully established.

KD is a diet with high fat (60–90%), low carbohydrate (2–5%), and moderate protein, which has been applied in the treatment of children with refractory epilepsy for decades (Sampaio 2016). The core of KD is high fat and low carbohydrate. Fat undergoes β-oxidation through the liver to generate BHB, acetoacetate, and acetone, of which BHB accounts for approximately 70% of the total ketone bodies. Studies have shown that BHB at physiological doses can inhibit the proliferation and metastasis of human CRC. A study confirmed that BHB acts through the surface receptor Hcar2 and induces the transcriptional regulator Hopx to change gene expression and inhibit CRC cell proliferation (Dmitrieva-Posocco et al. 2022). Furthermore, BHB increased the therapeutic effects of radiotherapy and chemotherapeutic drugs on human CRC cell lines (Hanna et al. 2023). Accordingly, this study aimed to analyze the growth inhibition effect of BHB on CRC-Oxa-resistant cells. Our data suggested that BHB could reverse the acquired resistance of CRC-resistant strains to Oxa. In addition, this study also established the CRC subcutaneous tumor model and metastasis model by subcutaneous injection or portal vein injection of 8 × 10^6^ HCT116-Oxa cells into mice. The results confirmed that the Oxa + BHB combination treatment group further inhibited the tumor growth of CRC and the number of liver metastatic nodules, suggesting that BHB can resensitize CRC to Oxa in vivo.

At present, the mechanism of Oxa resistance has not yet been fully elucidated. To explore the role of BHB in Oxa resistance, the common reasons are the upregulation of drug transport pumps such as P-gp, BCRP, and MRP, the NER overexpression, the cellular enzyme changes such as cytochrome P450 and glutathione-S-transferase, EMT, and tumor internal environment changes, among others (Riddell 2018). Moreover, the role of epigenetic mechanisms that do not involve gene sequence changes in tumor drug resistance has been increasingly concerning and has become a hot topic in tumor research. Various studies have reported that targeted histone modification can improve drug resistance in tumors (Furukawa and Kikuchi 2016; Bajbouj et al. 2021). Jiang et al. (Jiang et al. 2023). confirmed that the elevation of H3K14ac at the locus promoted the transcription and secretion of heat shock protein 70 in CRC cells, thereby promoting the tumorigenesis process. Sun et al. (Sun et al. 2019). suggested that acetyltransferase protein levels (P300 and GCN5) and H3K14ac levels were upregulated in acquired paclitaxel-resistant ovarian cancer cells. Moreover, studies have confirmed that CBP/p300 inhibitors and degraders could downregulate oncogene transcription by reducing H3K27ac, thereby overcoming drug resistance and inhibiting tumor progression (Chen et al. 2022). H3K79 is a lysine site located on the surface of the core sphere of histones. Relevant studies have reported that H3K79 methylation is involved in multiple physiological processes, as well as tumor occurrence and development. Zhang et al. (Zhang et al. 2014). showed that inhibition of histone H3K79 methylation can selectively inhibit the proliferation, self-renewal, and metastasis potential of breast cancer. Kim et al. (Kim et al. 2012). reported that H3K79 is upregulated in lung cancer cell lines and tumor tissues of patients with lung cancer, indicating that H3K79 methylation is a key histone modification that regulates cell proliferation and maybe a new histone marker for cancer. Kryczek et al. (Kryczek et al. 2014). suggested that high DOT1L and H3K79me2 expressions in the CRC tissue are predictive factors for low patient survival. Kari et al. (Kari et al. 2019). found that a small molecule inhibitor of H3K79me methyltransferase DOT1L combined with chemotherapeutic agents for the treatment of CRC displayed a therapeutic additive effect. Further, examination of H3K79me levels in patients with CRC revealed that lower levels were associated with a worse prognosis. Lv et al. (Lv et al. 2019). reported that miR-133b, as a key regulator, regulated the H3K79 methylation, thereby indirectly regulating a group of drug-resistant genes. Additionally, inhibiting or silencing the DOT1L expression could reverse the 5-fluorouracil and Oxa resistance of CRC cells. A previous study reported that H3K9me confers CRC 5-fluorouracil resistance by suppressing the Fas expression (Paschall et al. 2015). As H3K79me, H3K27ac, H3K14ac, and H3K9me expressions have been reported to contribute to the malignant phenotypes of CRC, this study used IHC to examine the expression levels of H3K79me, H3K27ac, H3K14ac, and H3K9me in tumor tissues of CRC patients with and without Oxa resistance. The results indicated that H3K79me, H3K27ac, and H3K14ac were significantly overexpressed in the tumor tissues of drug-resistant patients., This suggested that the elevated H3K79me, H3K27ac, and H3K14ac expression levels were indeed involved in the Oxa resistance mechanism of CRC.

Next, we further discovered that H3K79me and H3K27ac expressions in tumor tissues of patients with CRC were prominently negatively correlated with BHB. Moreover, BHB intervention downregulated H3K79 methylation and H3K27 acetylation levels in HCT116-Oxa and LoVo-Oxa cells in a dose-dependent manner. Thus, we speculate that BHB may resensitize CRC cells to Oxa depending on H3K79 methylation or H3K27 acetylation inhibition. Previous studies have reported that BHB can restrain histone methylation. Furthermore, it exhibits strong deacetylase activity. Wang et al. (Wang et al. 2021). suggested that BHB acts as an epigenetic regulator in histone methylation and acetylation to delay various age-related diseases. Castro et al. (Castro et al. 2021). found that the BHB diet did not affect the progression of aortic H3K27ac levels in atherosclerotic mice. In this study, HCT116-Oxa cells were treated with H3K79 methyltransferase DOT1L knockdown or H3K27ac inhibitor. Our study revealed that BHB treatment based on DOT1L knockdown had no significant effect on the proliferation, invasion, apoptosis, and EMT of HCT116-Oxa cells; however, the activity behavior of HCT116-Oxa cells treated with BHB after inhibiting H3K27 acetylation was strikingly suppressed. Inhibition of H3K79 methylation may lead to the lack of a target for BHB, making it unable to play its role. Therefore, the results suggested that BHB resensitized CRC cells to Oxa mainly by inhibiting H3K79 methylation rather than H3K27 acetylation.

Notably, in the subsequent in vivo study, BHB also enhanced the efficacy of Oxa in the context of without H3K79me. However, this promotion was significantly weaker than that in the context of H3K79me. IHC data indicated that BHB caused a significant suppression in H3K79me of transplanted tumor tissues. Moreover, observing H3K79me in transplanted tumor tissues of mice was challenging based on DOT1L knockdown whether injection of BHB or not, which confirmed that DOT1L knockdown successfully caused the blockage of H3K79me. These data suggested that BHB could enhance the efficacy of Oxa by suppressing H3K79me but not in a dependent manner in vivo, which was not consistent with our in vitro study. A recent study reported that the BHB administration altered the energy metabolism to increase ROS levels, thereby enhancing the efficacy of Oxa on CRC organoids (Sever et al. 2023). This study may explain the inconsistency between our in vitro and in vivo results. Since the H3K79 has been identified as one of the identified β-hydroxybutyrylation sites of histone by BHB (Bannister and Kouzarides 2011), we speculated that BHB suppression on the H3K79 methylation is caused by β-hydroxybutyrylation of H3K79, which will be explored in a future study.

The above results provide an experimental basis for the clinical research of BHB and identify an informative treatment strategy for patients with acquired drug resistance who have limited available treatment. Although some research achievements have been made in this area, deficiencies and problems remain unresolved. The regulatory mechanism of BHB inhibiting H3K79 methylation has not been elaborated. Moreover, the expression of P-gp and other drug resistance-related proteins will be further detected by IHC in the follow-up study. In conclusion, this study explained the mechanism of drug resistance and the evolution of CRC from the perspective of epigenetics and explored a new treatment method for drug resistance treatment of CRC.

## Conclusions

In summary, this article illustrates that elevated methylation of H3K79 may be a reason for the resistance of CRC cells to Oxa. This study proposes a strategy of using BHB combined with Oxa treatment to overcome the resistance and enhance the antitumor activity of BHB.

### Electronic supplementary material

Below is the link to the electronic supplementary material.

Supplementary Material 1

Supplementary Material 2

Supplementary Material 3

Supplementary Material 4

## Data Availability

The data that support the findings of this study are available from the corresponding author upon reasonable request.

## Abbreviations

BHB: β-hydroxybutyrate
Oxa: Oxaliplatin
CRC: Colorectal cancer
EMT: Epithelial–mesenchymal transition
H3K79me: H3K79 methylation
H3K27ac: H3K27 acetylation
KD: Ketogenic diet
