# Supplementary figures and images for: β-hydroxybutyrate resensitizes colorectal cancer cells to oxaliplatin by suppressing H3K79 methylation in vitro and in vivo
Source: Mol Med. 2024 Jun 23;30:95. doi: 10.1186/s10020-024-00864-1 (PMC11194918; doi:10.1186/s10020-024-00864-1)

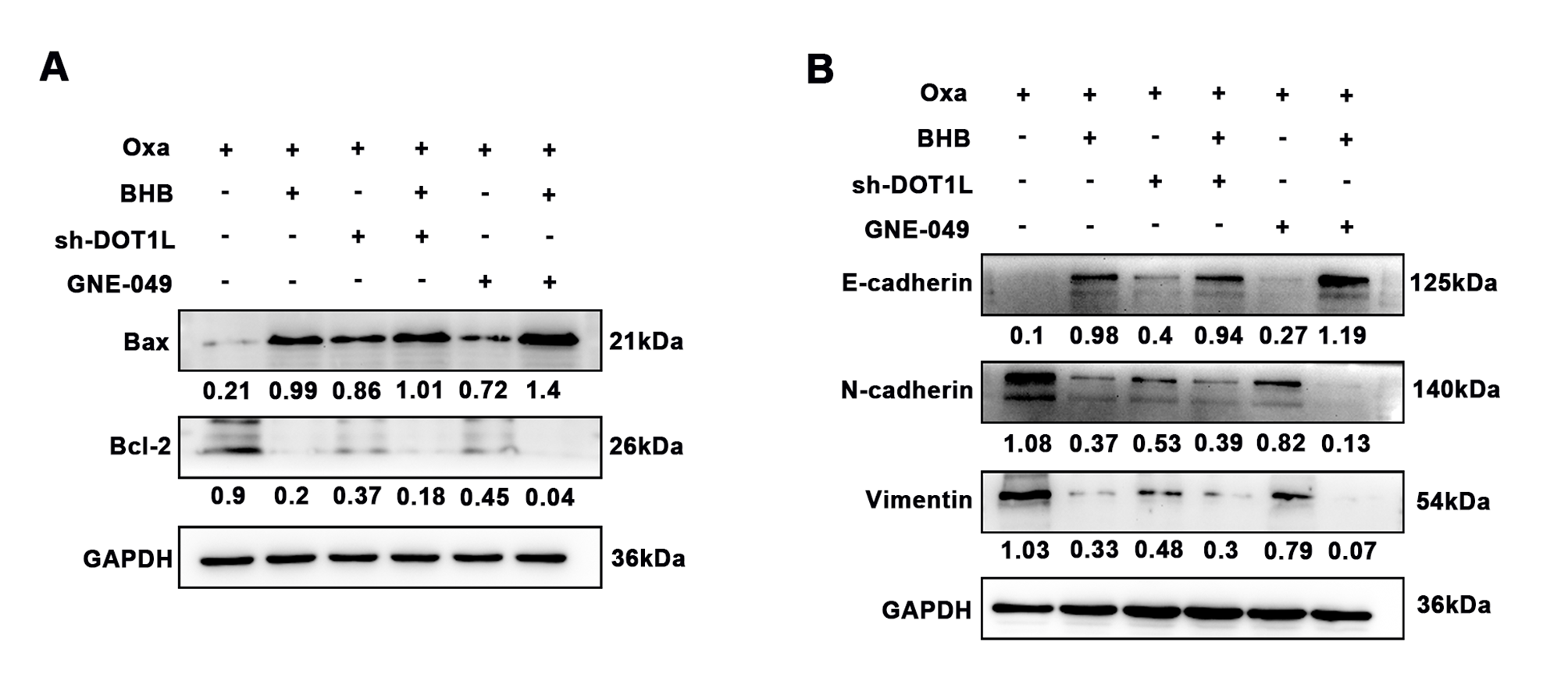

Supplement: Supplementary file 1 — Supplementary Material 1 [file 10020_2024_864_MOESM1_ESM.png]

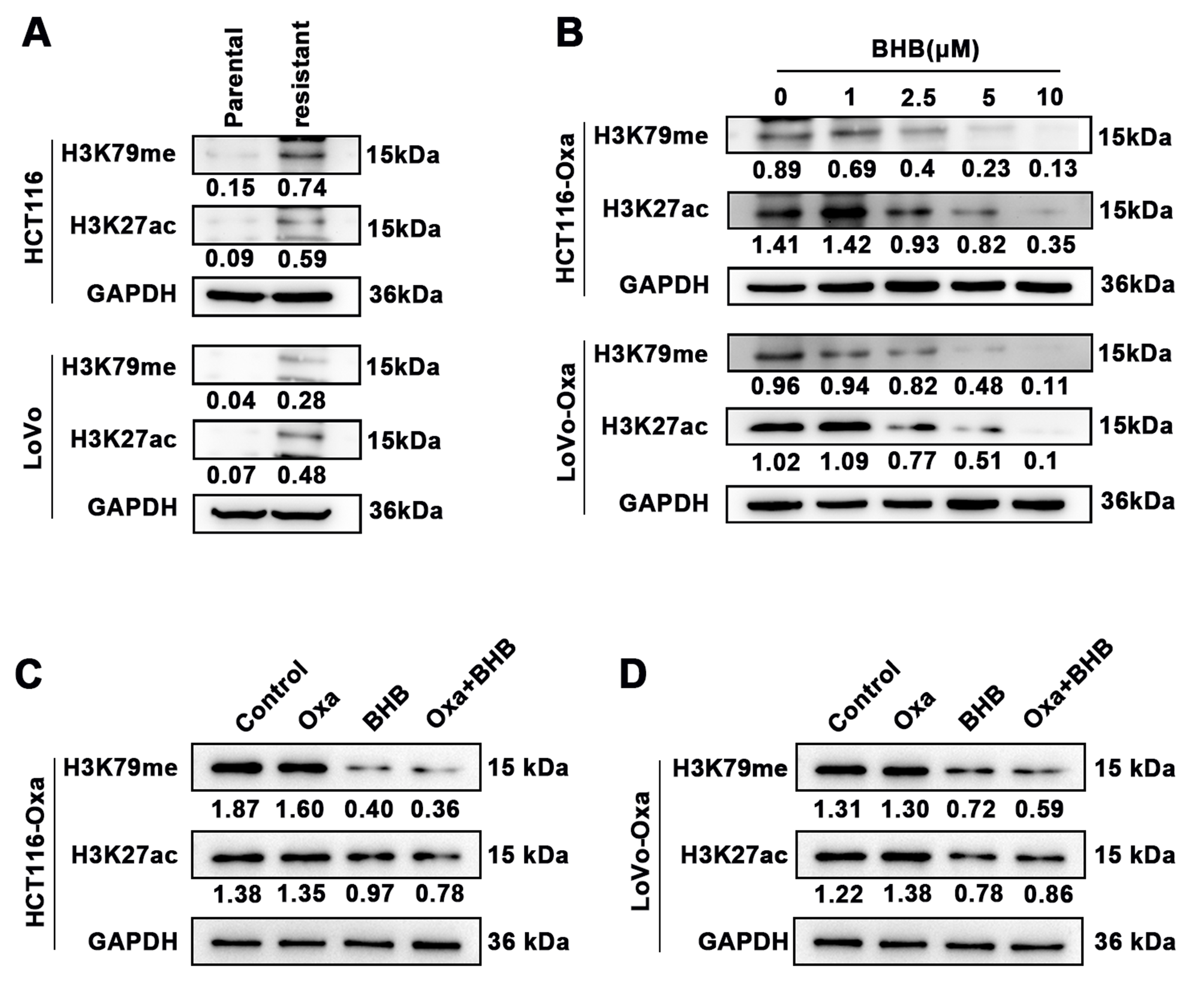

Supplement: Supplementary file 3 — Supplementary Material 3 [file 10020_2024_864_MOESM3_ESM.png]
