# Supplementary material for: β-hydroxybutyrate resensitizes colorectal cancer cells to oxaliplatin by suppressing H3K79 methylation in vitro and in vivo
Source: Mol Med. 2024 Jun 23;30:95. doi: 10.1186/s10020-024-00864-1 (PMC11194918; doi:10.1186/s10020-024-00864-1)
Supplement: Supplementary file 2 — Supplementary Material 2 [file 10020_2024_864_MOESM2_ESM.docx]

**Table S1. Primer sequence (5’-3’)**

| Gene | Forward Primer | Reverse Primer |
| --- | --- | --- |
| DOT1L | CTGCCGGTCTACGATAAACATC | AGCTTGAGATCCGGGATTTCT |
| GAPDH | TGTAGGCTCATTTGCAGGGG | TCCCATTCCCCAGCTCTCAT |

**Figure S1. BHB suppressed H3K79 methylation and H3K27 acetylation levels in CRC-resistant cells. A**: Western blotting determination of H3K79 and H3K27 protein levels in HCT-116-Oxa and LoVo-Oxa cells. **B**: Western blotting determination of protein levels of H3K79 and H3K27 in HCT-116 and LoVo parental and drug-resistant cells treated with Oxa at a series of concentrations (0, 1, 2.5, 5, and 10 μM) for 24h. Data: Mean ± SEM (n = 3).

**Figure S2. BHB facilitates apoptosis and EMT progression of CRC-resistant cells by inhibiting H3K79 methylation**

**A**: Western blotting determination of protein levels of Bax and Bcl-2 in HCT-116-Oxa cells, which inhibited H3K79 methylation, H3K27 acetylation, and/or BHB intervention. **B**: Western blotting determination of E-cadherin, N-cadherin, and vimentin protein levels in HCT-116-Oxa cells, which inhibited H3K79 methylation, H3K27 acetylation, and/or BHB intervention (n = 3).
