# Supplementary material for: β-hydroxybutyrate resensitizes colorectal cancer cells to oxaliplatin by suppressing H3K79 methylation in vitro and in vivo
Source: Mol Med. 2024 Jun 23;30:95. doi: 10.1186/s10020-024-00864-1 (PMC11194918; doi:10.1186/s10020-024-00864-1)
Supplement: Supplementary file 4 — Supplementary Material 4 [file 10020_2024_864_MOESM4_ESM.pdf]

www.enago.cn

www.voxtab.com

CERTIFICATE OF EDITING

This is to certify that the paper titled **β-hydroxybutyrate resensitizes colorectal cancer cells to oxaliplatin by suppressing H3K79 methylation in vitro and in vivo** commissioned to us by **Meng Deng, Peijie Yan, Hui Gong, and Jianjie Wang** has been edited for English language, grammar, punctuation, and spelling by Enago, an editing brand of Crimson Interactive Consulting Co. Ltd.

commisoned

ISO/IEC 27001 Information Security Management

ISO/IEC 27001:2013 Certified

ISO 9001 Quality Management

ISO 9001:2015 Certified

Issued by:

CRIMSON INTERACTIVE CONSULTING CO.,LTD 克里门森互动(北京)顾问有限公司 1101030093948

北京市海淀区中关村南大街甲6号铸诚大厦607室， 邮编100086
Tel: +86-10-5158 1987

Disclaimer: The author is free to accept or reject our changes in the document after our editing. However, we do not bear responsibility for revisions made to the document after our edit on **13th April, 2024.**

English	www.enago.com, www.voxtab.com, www.ulatus.com
Japan	www.enago.jp, www.voxtab.jp, www.ulatus.jp
Brazil	www.enago.com.br
Germany	www.enago.de
Turkey	www.enago.com.tr
China	www.enago.cn
Korea	www.enago.co.kr
Taiwan	www.enago.tw

About Crimson:
Crimson Interactive Consulting Co. Ltd. provides English language editing, transcription, and translation services to individuals and corporate customers worldwide.
